# Neonatal Bradypnea as an Under-Recognized Manifestation of Prader-Willi Syndrome: A Case Report

**DOI:** 10.7759/cureus.103241

**Published:** 2026-02-08

**Authors:** Ankit Ranjan, Shams Karim, Jeoffrey Jay Valentin, Sofia Fakih

**Affiliations:** 1 Department of Pediatrics and Neonatology, Medeor Hospital, Dubai, ARE

**Keywords:** dna-methylation, neonatal hypotonia, neonatal respiratory pathologies, prader-willi syndrome, preterm neonate

## Abstract

Prader-Willi syndrome (PWS) is a rare neurogenetic disorder caused by loss of expression of paternally inherited genes within the imprinted 15q11.2-q13 region. Diagnosis during the neonatal period is difficult, as symptoms are often reported in older infants or in childhood. Neonatal hypotonia, poor suck leading to feeding difficulties, and genital hypoplasia are commonly recognized features, but respiratory manifestations have been rarely described and may delay early diagnosis. We report a late-preterm neonate born at 36 weeks’ gestation with severe asymmetric intrauterine growth restriction who developed respiratory distress soon after birth, initially attributed to early-onset pneumonia. Despite resolution of sepsis, the infant demonstrated recurrent apnea followed by sustained bradypnea episodes beyond the expected course for gestational age. This was accompanied by marked hypotonia and poor feeding due to impaired suck-swallow-breathing coordination. Neuroimaging and metabolic investigations were unremarkable, prompting further evaluation for central causes of hypoventilation. Targeted whole-exome sequencing with copy-number analysis identified a pathogenic deletion involving the PWS critical region on chromosome 15q11.2-q13, and confirmatory DNA methylation analysis demonstrated loss of the paternal allele, establishing the diagnosis. With supportive care, the respiratory status gradually improved, and the infant was referred for multidisciplinary follow-up, including physiotherapy and endocrine evaluation. This case highlights neonatal bradypnea as a rare and under-recognized early manifestation of PWS and emphasizes the importance of considering this diagnosis in neonates with unexplained respiratory dysregulation and hypotonia to facilitate timely genetic testing and appropriate multidisciplinary management.

## Introduction

Prader-Willi syndrome (PWS) is a rare genetically determined multisystem disorder caused by loss of expression of paternally inherited genes within the imprinted 15q11.2-q13 chromosomal region [1,2]. The estimated prevalence ranges from approximately 1 in 10,000 to 1 in 30,000 live births, although the true incidence is likely underestimated due to under-recognition in early life [3]. First described in 1956, PWS remains frequently undiagnosed during the neonatal period despite advances in molecular diagnostics [4].

In most cases, the diagnosis is established later in infancy or early childhood, when hyperphagia, excessive weight gain, and characteristic behavioural features become evident [5]. Delayed recognition at this stage limits opportunities for early intervention and structured care, which are known to influence long-term outcomes. The neonatal phenotype of PWS is typically characterized by severe hypotonia, reduced spontaneous activity, poor suck, and feeding difficulties, often leading to early growth failure [5,6]. However, these features are nonspecific and overlap considerably with those seen in preterm infants or neonates affected by hypoxic, metabolic, or central nervous system disorders [6]. As a result, PWS may not be considered in the initial differential diagnosis, particularly when prematurity or intrauterine growth restriction (IUGR) provides an alternative explanation for hypotonia and feeding difficulties.

Several cohort studies have identified antenatal findings such as reduced fetal movements and polyhydramnios, along with postnatal features including genital hypoplasia and subtle craniofacial characteristics as early indicators of PWS [7]. However, recognition of these features remains inconsistent, and neonatal diagnosis is still uncommon. Early confirmation of PWS in the neonatal period has important clinical implications, as it enables timely multidisciplinary involvement, targeted feeding strategies, early physiotherapy, planned endocrine evaluation as the child grows, and appropriate genetic counselling with psychosocial support for families during a critical period of early caregiving.

PWS is associated with hypothalamic and autonomic dysfunction, which has been implicated in abnormalities of ventilatory control, sleep-related breathing disorders, and impaired responses to hypoxia and hypercapnia later in life. However, the relevance of these mechanisms to respiratory regulation in the neonatal period remains incompletely understood. 

We report the case of a late-preterm neonate who presented with initial respiratory distress followed by recurrent apnea with sustained bradypnea, along with generalized hypotonia in the immediate postnatal period. The diagnosis of PWS was confirmed by DNA methylation analysis, the current gold-standard diagnostic test for PWS. This case highlights an uncommon neonatal presentation of PWS and underscores the importance of considering this diagnosis in neonates with unexplained respiratory dysregulation and hypotonia disproportionate to gestational age.

## Case presentation

A late-preterm neonate was born at 36 weeks of gestation to a second-gravida mother with a very low birth weight of 1320 grams. Antenatal history was suggestive of gestational diabetes mellitus controlled with dietary modifications. There was no history of polyhydramnios or perceived reduction in fetal movements. However, severe asymmetric IUGR in the fetus was noted in serial ultrasound scans, and an increased systolic-to-diastolic ratio in the umbilical artery Doppler at 34 weeks necessitating lower-segment caesarean section at 36 weeks. There was evidence of premature prolonged rupture of membranes (PPROM) for 22 hours prior to delivery. The baby had a smooth perinatal transition with Apgar scores of 8 and 9 at 1 and 5 minutes, respectively.

Birth weight and length were below the 10th percentile, while head circumference was at the 30th percentile as per the modified Fenton's growth chart, with a ponderal index of 1.7 g/cm³, consistent with asymmetric IUGR. On head-to-toe examination, no major craniofacial dysmorphism was apparent; however, genital hypoplasia was noted in the form of a hypoplastic scrotum with bilateral undescended testes. Neurological examination demonstrated generalized hypotonia with predominant axial involvement, reduced spontaneous movements, and marked head lag. Limb tone was reduced but symmetric with no evidence of focal neurological deficit.

**Figure 1:**
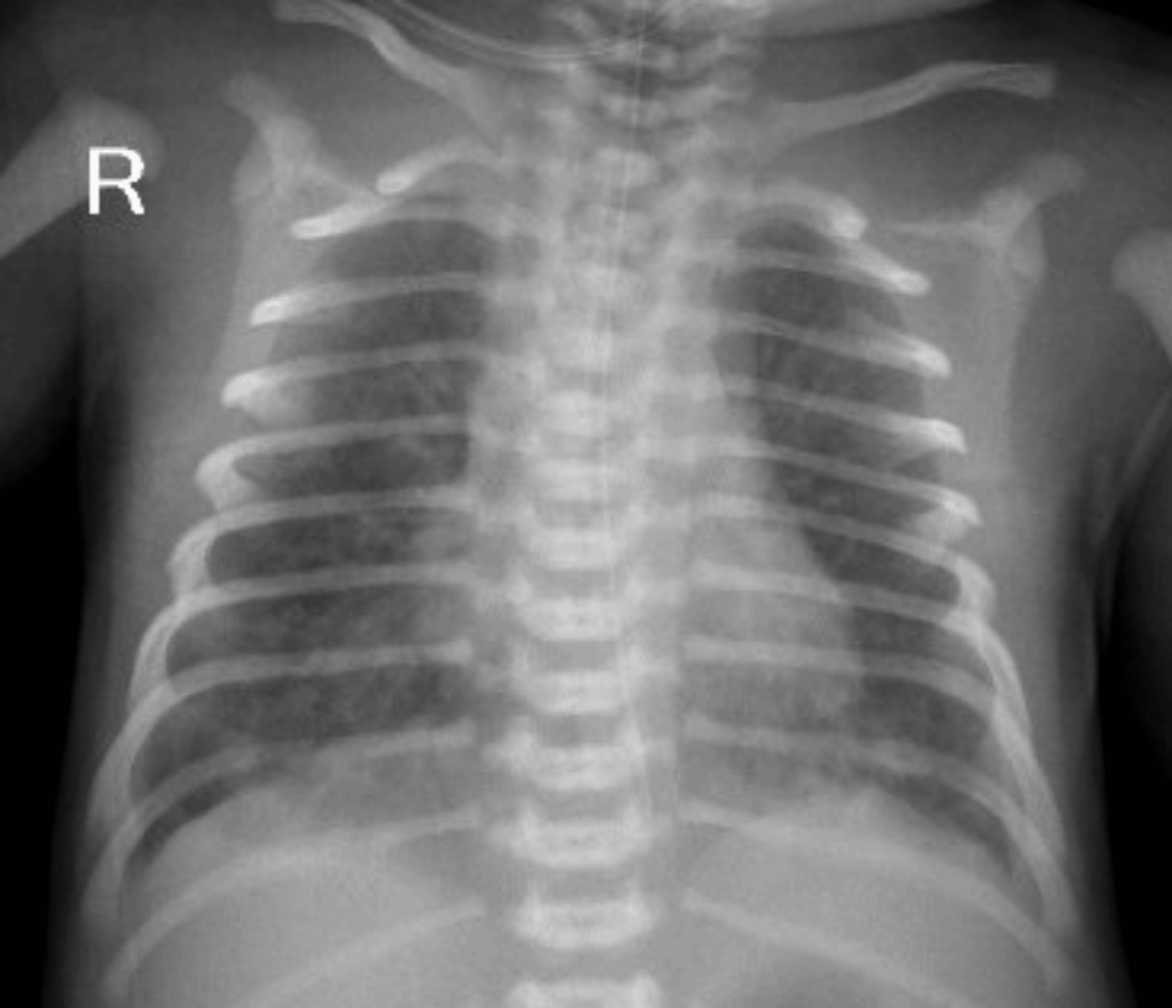
Chest radiograph obtained in the early neonatal period Anteroposterior chest radiograph obtained within the first 24 hours of life demonstrating bilateral patchy pulmonary opacities, more prominent in the perihilar and lower lung zones consistent with parenchymal disease. These findings were interpreted in the clinical context as suggestive of early-onset congenital pneumonia.

The baby had respiratory distress since birth with compensated respiratory acidosis (pH 7.29, pCO₂ 55.3), requiring high-flow nasal cannula (HFNC) support. Chest radiography demonstrated bilateral patchy pulmonary opacities suggestive of multifocal consolidations (Figure 1). In the setting of PPROM, respiratory distress, elevated C-reactive protein (CRP), and the requirement for respiratory support, early-onset sepsis with congenital pneumonia was suspected. The infant was started on ampicillin and amikacin, with improvement in sepsis markers and resolution of respiratory distress within the next 72 hours. 

The infant developed an episode of central apnea at 90 hours of life requiring tactile stimulation, prompting initiation of intravenous caffeine citrate (20 mg/kg) followed by daily maintenance doses of 5 mg/kg. Metabolic work-up ruled out correctable causes, with normal electrolytes and no evidence of intraventricular hemorrhage or apparent brain malformation on neurosonography. Between 72 hours of life and the onset of apnea, the infant’s baseline resting respiratory rate ranged between 30 and 35 breaths per minute. Despite initial stabilization, a second significant apnea episode occurred on day 10, associated with persistent bradypnea requiring escalation of HFNC flow. Between day 10 and day 17 of life, the infant consistently demonstrated sustained bradypnea with resting respiratory rates ranging between 22 and 30 breaths per minute, with recorded nadirs of 20-22 breaths per minute, persisting beyond 37 weeks’ corrected gestational age. The nadirs were noted especially during sleep. Despite the low respiratory rates, oxygen saturation remained stable on HFNC flow of 5 liters per minute with no additional oxygen requirement. Common late-preterm causes of apnea and bradypnea were systematically considered and excluded during the clinical course. Hemoglobin levels remained within acceptable limits for age, making anemia unlikely. Feeding-related aspiration or gastroesophageal reflux was considered; however, bradypnea and apnea episodes were not temporally associated with feeds. There was no clinical evidence of aspiration, and chest findings resolved early in the course. Medication exposure was also reviewed. Aside from caffeine citrate, no sedatives, opioids, or respiratory depressants were administered. Persistent infection was also considered unlikely as inflammatory markers normalized, cultures remained sterile, and there were no ongoing clinical signs of sepsis. Serial blood gas analyses showed no evidence of sustained hypercapnia as the baby was supported with HFNC flow. Blood gas carbon dioxide values remained within acceptable limits for age. These findings suggested impaired respiratory drive rather than primary lung disease or neuromuscular failure.

In view of the persistent bradypnea despite treatment of early-onset sepsis, hypotonia, and IUGR status, a targeted evaluation for congenital central hypoventilation syndrome (CCHS) and neuromuscular disorders was initiated with exome sequencing. Respiratory support was gradually tapered and discontinued on day 21 of life, as the respiratory rates improved to around 30-35 breaths per minute with no evidence of hypercapnia on serial blood gas analysis.

The nutrition was initially managed on oro-gastric feeding as oral feeding progression was complicated by significant feeding difficulties and persistent hypotonia. Oral feeding trials were initiated after three weeks of life following the removal of respiratory support. These attempts revealed poor suck-swallow-breathing coordination, necessitating supervised oral feeds and oro-motor stimulation.

Targeted whole-exome sequencing with copy-number analysis identified a 4.96-megabase deletion on chromosome 15q11.2-q13, encompassing 15 protein-coding genes. Among these, seven were disease-associated genes within the Prader-Willi/Angelman critical region (*GABRA5*, *GABRB3*, *HERC2*, *MAGEL2*, *MKRN3*, *OCA2*, and *UBE3A*). Although the exact breakpoints could not be determined, the deletion clearly involved the imprinted 15q11-q13 region.

**Figure 2:**
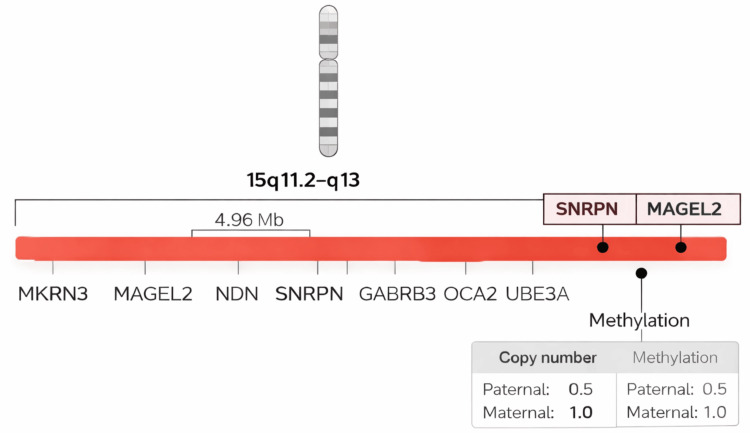
Molecular basis of Prader-Willi syndrome in the index case Schematic representation of chromosome 15 highlighting the imprinted 15q11.2-q13 region. Whole-exome sequencing with copy-number analysis identified an approximately 4.96 Mb heterozygous deletion encompassing key genes within the Prader-Willi syndrome critical region, including *MKRN3*, *MAGEL2*, *NDN*, *SNRPN*, *GABRB3*, *OCA2*, and *UBE3A*. Methylation-specific analysis proved diagnostic demonstrating loss of the paternally inherited allele, with a copy-number ratio of 0.5 and abnormal methylation at the SNRPN and MAGEL2 loci, confirming the molecular diagnosis of Prader-Willi syndrome.

Because this region is imprinted, the clinical outcome depends on the parental origin of the deleted chromosome. Whole-exome sequencing confirmed the deletion but could not determine parental origin or methylation status; therefore, confirmatory methylation testing was performed. Methylation-specific multiplex ligation-dependent probe amplification demonstrated heterozygous deletion of several genes within the PWS region (*MKRN3*, *MAGEL2*, *NDN*, *SNRPN*, *OCA2*, *GABRB3*, *ATP10A*, and *UBE3A*). Aberrant methylation of the normally paternally expressed *SNRPN* and *MAGEL2* genes was observed, with a pattern consistent with 100% methylation of the paternal allele and a copy number ratio of 0.5, confirming loss of the paternal 15q11-q13 region as shown in Figure 2. 

Together, these findings established the diagnosis of PWS, explaining the infant’s clinical presentation, including neonatal hypotonia, poor feeding, recurrent bradypnea suggestive of impaired central respiratory control, and early growth failure. Such sustained bradypnea episodes have rarely been described in PWS cohorts. The infant was referred for physiotherapy for early stimulation, pediatric endocrinology consultation for planning of hormone replacement therapy if indicated, and pediatric surgical follow-up for management of cryptorchidism.

## Discussion

PWS is a complex neurogenetic disorder resulting from loss of expression of paternally inherited genes within the imprinted chromosome 15q11.2-q13 region. While the classical phenotype is well recognized, diagnosis is frequently delayed, particularly when early manifestations are subtle or atypical. This is especially true in the neonatal period, where hypotonia and feeding difficulties are often attributed to prematurity, IUGR, or intercurrent illness. Respiratory abnormalities, when present, may further obscure the underlying diagnosis. The present case draws attention to an uncommon neonatal presentation of PWS in which recurrent bradypnea and apnea dominated the early clinical course, raising concern for impaired central regulation of breathing.

Disordered breathing is a well-recognized feature of PWS later in life. Studies have documented central sleep apnea, obstructive sleep apnea, and hypoventilation in infants and children with PWS, reflecting impaired ventilatory responses to hypoxia and hypercapnia and underlying hypothalamic dysfunction [8-10]. However, most of these observations relate to older infants and children, and primary neonatal respiratory dysregulation has received comparatively little attention. When respiratory issues are described in neonates with PWS, they are more commonly attributed to aspiration, airway obstruction secondary to hypotonia, or intercurrent infection rather than intrinsic abnormalities of respiratory control [7].

In the present case, persistent bradypnea and recurrent apnea were observed even after resolution of early-onset pneumonia, with normal neurosonography and no evidence of ongoing hypercapnia. This pattern raised concern for a central cause of hypoventilation, prompting consideration of CCHS, a rare disorder of autonomic respiratory control [11]. However, several features argued against this diagnosis, including the absence of sustained hypercapnia, lack of autonomic instability, and gradual improvement with advancing postnatal age. Collectively, these findings were more consistent with a transient impairment of respiratory drive rather than a fixed ventilatory control disorder. In the absence of ongoing pulmonary disease, infection, anemia, medication exposure, or metabolic disturbance with the subsequent molecular diagnosis, these respiratory findings occurred in association with PWS and may reflect underlying hypothalamic or autonomic dysfunction, although a contributory effect of late prematurity cannot be excluded. 

Within the context of PWS, such respiratory instability is plausibly explained by hypothalamic and autonomic dysfunction, which are recognized contributors to abnormal respiratory regulation in affected individuals [10]. Importantly, this distinction suggests that respiratory dysregulation in neonatal PWS may improve with maturation and supportive care, in contrast to the lifelong ventilatory dependence typically associated with CCHS. The prominence of respiratory manifestations in this infant may also have been influenced by severe asymmetric IUGR. Pregnancies affected by PWS have been associated with abnormal antenatal findings such as reduced fetal movements, polyhydramnios, and placental insufficiency [12]. The combined effects of growth restriction, late prematurity, and underlying genetic dysfunction may have increased vulnerability of central respiratory control mechanisms, thereby unmasking an early and otherwise under-recognized manifestation of the syndrome.

A stepwise genetic approach was central to establishing the diagnosis in this case. Whole-exome sequencing with copy-number analysis identified a pathogenic deletion involving the PWS/Angelman critical region, while methylation-specific testing confirmed loss of the paternal allele, the most common molecular mechanism underlying classical PWS [13]. Early molecular confirmation avoided further invasive investigations and enabled timely redirection of care. Relevant published literature describing the neonatal and pediatric cohort of PWS with special emphasis on respiratory findings (if any) has been mentioned in Table 1. 

**Table 1:** Published studies describing neonatal and early-life features of Prader-Willi syndrome with emphasis on respiratory manifestations Comparison of published studies describing neonatal and early-life manifestations of Prader-Willi syndrome, with emphasis on respiratory findings. The present case is unique in demonstrating persistent neonatal bradypnea as a prominent early feature, preceding classical recognition of Prader-Willi syndrome. IUGR, intrauterine growth restriction; PWS, Prader-Willi syndrome; GH, growth hormone.

Study	Study design	Population	Key neonatal findings	Respiratory manifestations	Key relevance
Hu et al., 2021 [7]	Case series + literature review	7 neonates with genetically confirmed PWS	Severe hypotonia, poor feeding, reduced fetal movements, genital hypoplasia	Respiratory distress common; apnea reported but not primary focus	Highlights early neonatal presentation of PWS but limited emphasis on persistent bradypnea
Festen et al., 2006 [8]	Observational study	Prepubertal children with PWS	Hypotonia, obesity, GH therapy effects	High prevalence of sleep-related breathing disorders	Demonstrates impaired respiratory regulation in PWS beyond neonatal period
Miller et al., 2009 [9]	Prospective pilot study	Infants with PWS receiving GH therapy	Hypotonia, feeding difficulties	Sleep-disordered breathing observed early in infancy	Supports early-life vulnerability of respiratory control in PWS
Cataldi et al., 2021 [10]	Narrative review (human + animal data)	Children and adults with PWS	Hypothalamic dysfunction	Central and obstructive sleep-disordered breathing	Provides mechanistic insight into abnormal respiratory regulation in PWS
Grootjen et al., 2022 [12]	Retrospective cohort study	Children with PWS	Reduced fetal movements, hypotonia, feeding difficulties	Respiratory issues reported but not dominant	Robust prenatal-neonatal phenotype data; respiratory findings under-represented
Present case	Single case report	Late-preterm neonate with PWS	Severe hypotonia, feeding failure, asymmetric IUGR	Persistent neonatal bradypnea and apnea mimicking central hypoventilation	Highlights bradypnea as a rare, early, and under-recognized manifestation of neonatal PWS

Early diagnosis of PWS allows planned endocrine evaluation, including consideration of growth hormone therapy, which has been shown to improve body composition, linear growth, and neurodevelopmental outcomes [14]. In addition, structured health supervision and anticipatory guidance are essential components of long-term care for individuals with PWS [15].

In the absence of ongoing pulmonary disease, infection, anemia, medication exposure, or metabolic disturbance, the respiratory symptoms raised concern for impaired respiratory drive. Given the subsequent molecular diagnosis, these respiratory findings occurred in association with PWS and may reflect underlying hypothalamic or autonomic dysfunction, although a contributory effect of late prematurity cannot be excluded.

This case contributes to the existing literature by describing a neonate with molecularly confirmed PWS who exhibited recurrent bradypnea and apnea during the neonatal period. While respiratory dysregulation has been reported in older infants and children with PWS, persistent neonatal bradypnea has been infrequently emphasized in published neonatal cohorts and may be under-recognized, particularly in preterm or growth-restricted infants [7]. In the present case, recurrent bradypnea occurred in association with generalized hypotonia and feeding difficulty and persisted beyond the expected maturational period, prompting further evaluation and genetic testing. Increased awareness of this respiratory phenotype may support earlier consideration of PWS in neonates with unexplained respiratory dysregulation and hypotonia, facilitating timely diagnosis and multidisciplinary care.

## Conclusions

This case highlights an under-recognized neonatal presentation of PWS, in which persistent bradypnea accompanied generalized hypotonia and feeding difficulty. While respiratory instability in late-preterm infants is often attributed to immaturity, persistence beyond the expected maturational period should prompt consideration of alternative diagnoses. In neonates presenting with hypotonia, feeding difficulties, and unexplained persistent bradypnea, early evaluation for PWS with DNA methylation testing remains the gold standard and may facilitate timely diagnosis, appropriate multidisciplinary care with anticipatory guidance for families.
